# Medical Ethics

**Published:** 1844-02

**Authors:** 


### MEDICAL ETHICS.

Professor Harrison, of Cincinnati, has favored the profession with an excellent lecture on the subject of *Medical Ethics.* The profession needs many such lectures. They ought to be delivered all over the country. We ought to have line upon line—precept upon precept. The good lessons ought to be repeated continually. Dr. Harrison's lecture would form a good manual on the *duties of physicians.* We hope it will be circulated widely, for we are sure the principles so happily enforced in it cannot be contemplated without profit. Y.

